# Telocytes’ Role in Modulating Gut Motility Function and Development: Medical Hypotheses and Literature Review

**DOI:** 10.3390/ijms23137017

**Published:** 2022-06-24

**Authors:** Daniel Dumitru Banciu, Dragoș Crețoiu, Sanda Maria Crețoiu, Adela Banciu, Daniel Popa, Rodica David, Cristian Stefan Berghea-Neamtu, Calin Remus Cipaian, Mihai Octavian Negrea, Mihaela Gheonea, Bogdan Neamtu

**Affiliations:** 1 Department of Bioengineering and Biotechnology, Faculty of Medical Engineering, Polytechnic University of Bucharest, 011061 Bucharest, Romania; danieldumitrubanciu@gmail.com (D.D.B.); adela.banciu79@gmail.com (A.B.); 2 Department of Morphological Sciences, Cell and Molecular Biology and Histology, Carol Davila University of Medicine and Pharmacy, 050474 Bucharest, Romania; dragos@cretoiu.ro; 3 Fetal Medicine Excellence Research Center, Alessandrescu-Rusescu National Institute for Mother and Child Health, 050474 Bucharest, Romania; 4 Faculty of Medicine, “Lucian Blaga” University, 550024 Sibiu, Romania; danieliulian.popa@ulbsibiu.ro (D.P.); cristian.berghea@ulbsibiu.ro (C.S.B.-N.); calin.cipaian@ulbsibiu.ro (C.R.C.); dr.mihai.negrea@gmail.com (M.O.N.); bogdan.neamtu@ulbsibiu.ro (B.N.); 5 Institute for Research on the Quality of Society and the Sciences of Education, University Constantin Brancusi of Targu Jiu, Republicii 1, 210185 Targu Jiu, Romania; david.rodica.mba@gmail.com; 6 Research and Telemedicine Center for Neurological Diseases in Children, Pediatric Clinical Hospital Sibiu, 550166 Sibiu, Romania; 7 County Clinical Emergency Hospital of Sibiu, 2–4 Corneliu Coposu Str., 550245 Sibiu, Romania; 8 Department of Neonatology, University Emergency County Hospital, 200642 Craiova, Romania; drmgheonea@yahoo.com; 9 Department of Neonatology, University of Medicine and Pharmacy of Craiova, 200349 Craiova, Romania; 10 Faculty of Engineering, “Lucian Blaga” University of Sibiu, 550025 Sibiu, Romania

**Keywords:** telocytes, infant colic, inflammatory bowel diseases, Crohn’s disease, ulcerative colitis, irritable bowel syndrome

## Abstract

This review article explores the telocytes’ roles in inflammatory bowel diseases (IBD), presenting the mechanisms and hypotheses related to epithelial regeneration, progressive fibrosis, and dysmotility as a consequence of TCs’ reduced or absent number. Based on the presented mechanisms and hypotheses, we aim to provide a functional model to illustrate TCs’ possible roles in the normal and pathological functioning of the digestive tract. TCs are influenced by the compression of nearby blood vessels and the degree of fibrosis of the surrounding tissues and mediate these processes in response. The changes in intestinal tube vascularization induced by the movement of the food bowl, and the consequent pH changes that show an anisotropy in the thickness of the intestinal tube wall, have led to the identification of a pattern of intestinal tube development based on telocytes’ ability to communicate and modulate surrounding cell functions. In the construction of the theoretical model, given the predictable occurrence of colic in the infant, the two-layer arrangement of the nerve plexuses associated with the intestinal tube was considered to be incompletely adapted to the motility required with a diversified diet. There is resulting evidence of possible therapeutic targets for diseases associated with changes in local nerve tissue development.

## 1. Introduction

Telocytes (TCs) are interstitial cells with distinct features [1,2,3]. They have extensions that react to mechanical stimulation through calcium channels [4]. The ability of TCs to communicate with surrounding cells, especially stem cells (SCs), through gap junctions [5] and extracellular vesicles [6,7,8] opens a wide range of questions. Moreover, the hypothesis that TCs are capable of carrying out the cellular niche for stem cell regulation and support suggests that TCs could play an important part in the response to major changes in homeostasis. It also suggests that TCs have a significant functional role in tissues that have an increased turnover.

This article explores in a review the telocytes’ roles in inflammatory bowel diseases (IBD), presenting the mechanisms and hypotheses related to epithelial regeneration, progressive fibrosis, and dysmotility as a consequence of TCs’ reduced or absent number. Based on the presented mechanisms and hypotheses, we aim to provide a functional model to illustrate TCs’ possible roles in the normal and pathological functioning of the digestive tract. The digestive tract retains its basic structural layers along its path, despite the presence of significant functional changes from one area to another. In addition, the digestive tract presents important muscular and nervous components that sustain the movement of the fecal bowl. This, in correlation with the ability of TCs to react to mechanical stimuli through calcium as a second mediator, provides a perspective for understanding the maturation and functioning of digestive tract motility. This article first covers the implications of TCs’ functions in inflammatory bowel diseases, then details the model proposed for TC signaling and discusses the application of our model in intestinal colic, Crohn’s disease, ulcerative colitis, and irritable bowel syndrome in subsequent sections. Furthermore, possible novel treatment targets in these pathologies are discussed within each chapter.

## 2. TCs and Inflammatory Bowel Disease (IBD)

TCs represent a distinct category among stromal cells, which include pericytes, myofibroblasts, fibroblasts, vascular endothelial cells, macrophages, and dendritic cells. They have long protrusions (telopodes) consisting of alternating structures, namely podomers (thin segments) and podoms (dilated regions)—both of which contain endoplasmic reticulum, caveolae, and mitochondria [9]. A distinctive combination of CD34 and platelet-derived growth factor receptor α (PDGFRα) correlated with the absence of c-kit (CD117) seems to distinguish stromal telocytes in the GI tract from fibroblasts [10]. Compared to fibroblasts, TCs have distinct patterns of gene expression as well as micro-RNA and proteomic profiles, with important roles in neurotransmission, immunomodulation, extracellular matrix organization, angiogenesis, and regeneration. In addition, recent studies have revealed that both epithelial TCs and subepithelial TCs residing in the GI tract express specific receptors and transcriptional factors [11].

Telocytes distinguish themselves among the aforementioned cell types in GI layers by participating with interstitial Cajal cells (ICC) in the formation of 2D networks between the mucosal, submucosal, and circular muscle layers or 3D networks in the other muscle layers and the myenteric plexus. To date, TC subtypes have been described according to their location in the GI’s layers and the GI organ [12,13,14] as follows: (i.i) TCs—stem-cell-like (TCs-STML), including (i.ii) those transmitting the pace-maker activity, (i.iii) submucosal (TCs-SM) in the stomach and submucosa/circular layer, and (i.iv) subserosal (TC-SS) in the gut serosa/longitudinal muscle; (ii) septal (TC-SPT) in muscular bundles (along/around muscular fibers); (iii) generating slow-wave GI myenteric activity (TCs-MY) consisting of a network of multipolar pacemaker cells around the myenteric plexus; (iv) modulating the enteric neurotransmission-deep myenteric plexus (TCs-DMP), forming a network of multipolar cells in the proximity of nerve bundles; and (v) intramuscular (TCs-IM) with a bipolar aspect aligned along smooth muscle cells within the circular and longitudinal muscle layers.

Within the mucosal layer, TCs are involved in the transduction of sensory and immune signals, and in the maintenance of mucosal homeostasis [9,10]. Recently, mounting evidence highlights the role of TCs situated in the epithelia and lamina propria. Underlining the intestinal epithelium, TCs act as “nurse” cells for cryptal stem cells with an important role in tissue regeneration and repair [9,13,14,15,16]. The hypothesized mechanisms related to the epithelial cells’ integrity refer to: (i) an on/off programmed function at different moments in time; (ii) a localized response secondary to microbiome spatiality and nutrient gradients; and (iii) epithelial gene expression regulating the villus stem cells and telocytes. An impairment in these mechanisms might lead to inflammation in the mucosa and submucosa. Recent research reports on intestinal inflammatory diseases focus on these issues [16,17]. Epithelial TCs of the gastrointestinal tract markedly expressed Lgr 5+ (a leucine-rich-repeat-containing G-protein-coupled receptor 5) encoded by a gene previously categorized as specific for epithelial cryptal stem cells [16]. In addition, studies have shown that subepithelial TCs express Foxl1 (a winged helix transcription factor) and Gli1 (the hedgehog signaling mediator) as crucial stem cell proliferative triggers (using the Wnt pathway signals) with an essential role in the regeneration of the high-turnover epithelial cell line [13,14]. 

The stem cell compartment is located at the base of the crypts along with the Paneth cells. Stem cell behavior can be modulated by several signal pathways (Wnt, Hedgehog, Notch, BMP). These pathways seem to be crucial in inflammatory bowel diseases (IBD). Stem cells can differentiate into Paneth cells mainly by the Wnt signaling pathway through the TCF4 transcription factor which triggers the expression of several genes including alpha-defensins [17]. Paneth cells play a defensive role and clean the crypts by releasing antimicrobial molecules such as phospholipase A2, lysozyme, and most importantly, the alpha-defensins HD5 and HD6. Paneth cells play an important role against several virus strains, bacteria (Gram-positive/negative), and fungi [17]. In IBD, the reduction in TCF4 expression, leading to impaired Wnt signaling, is associated with a low level of expression of HD5 and HD6 in both Crohn’s disease (CD) and ulcerative colitis (UC). Conversely, beta-defensins seem to have a higher expression in IBD, particularly HBD1 in CD and HBD2 and HBD3 in UC [17].

TCs are connected with immune cells and endothelial cells by gap junctions and communicate in a paracrine fashion (exo/ectosomes). Hence, TCs seem to be involved in immunomodulatory effects, angiogenesis, and tissue regeneration by inhibiting oxidative stress and, consequently, cellular aging [9,10,15]. Although many research reports have brought forth evidence that the Wnt pathway is strongly connected to other inflammatory pathways and plays an important role in inflammation regulation, it is still unknown how it actually contributes to healing processes [18].

TCs along with fibroblasts organize and control the extracellular matrix (ECM) [9]. They confine the inflammatory cells in their meshes and also seem to play a supportive role in intestinal motility. The loss of TCs in inflammatory bowel diseases is associated with a higher number of fibroblasts and an increased but disorganized ECM output with progressive fibrosis [9,10,15].

A crucial role of TCs in the GI layers is related to facilitating neurotransmission and regulating gut motility by establishing a functional connection between: (i) the nerve endings in the submucosal plexus and myenteric plexus, (ii) the pacemakers of gut motility and neurotransmission (the ICC), and (iii) smooth muscles (SMC) [9,10,15]. Chronic inflammation and fibrosis in IBD lead to TCs’ sequestration with subsequent dysmotility [10]. 

Crohn’s disease (CD) and ulcerative colitis (UC) represent the main inflammatory bowel diseases (IBD) with an increasing worldwide incidence. IBD’s clinical presentation is a consequence of the chronic inflammation of the whole intestine with recurrent episodes triggering abdominal pain, bloody stools, and GI dysmotility. In CD, the inflammation affects all the GI layers and involves the whole GI tract, but the terminal ileum and/or the colon are predominantly affected. In UC, the inflammation leads to ulcers affecting both the mucosa and submucosa in the colon and rectum. In IBD, chronic inflammation eventually leads to mucosal loss and severe fibrosis with debilitating dysmotility which is typically more severe in CD. Multiple factors have been incriminated in IBD’s chronic inflammation; however, several independent factors have been found to be associated with its etiology [18]. Genetic susceptibility, epithelial line disruption, microbiome impairment, and subsequent invasion of the submucosa followed by a strong immune response have been described in the literature. The microbiome load concentration ranges from 10^7^–10^8^ organisms/gram in the distal ileum up to 10^11^–10^12^ organisms/gram in the luminal content of the colon. As a result, epithelial injury and subsequent pathogen influx trigger an enhanced immune response [17]. Although different phenotypes have been described for CD and UC, it seems to be innate immunity that is predominantly defective rather than adaptative, which relates to the mucosal barrier and its renewal capacity. Both stem cells and TCs play a crucial role in this respect by modulating the local response based on the adjacent microbiome architecture and nutrient gradients.

Moreover, recent reports highlight the progressive loss of TCs leading to a reduced number or even absence of TCs in fibrotic areas in both CD and UC, correlating to the severity of the disease. Consequently, TCs and ICC reduction have been proposed as key events in the dysmotility observed in IBD [9,10,15]. In the described context, understanding the TCs’ functionality becomes highly important to propose a theoretical model of TC signaling.

## 3. The Proposed Functional Model of TCs Signaling

Variables such as time, delay, and phase-shift can be utilized to describe the behavior of three-dimensional structures when subjected to a non-uniform or non-isotropic stimulus. In the intestinal tube, signaling can occur by means of mechanical stimuli (i.e., the movement of food) which exhibit a directional preference across the gastrointestinal tract. Simultaneously, chemical stimuli which mediate the different signaling pathways have the potential to create differentiated delays in accordance with local vascularization and its modulation and compression, as well as local fibrosis which may hinder the diffusion of signaling factors within the surrounding 3D structures. The disposition in the concentric vascular muscular and nervous plexus layers within the digestive tract enhances the anisotropy of the described signaling mechanisms and can allow for the development of subtle morphogenetic pathways. Understanding these pathways may lead to new therapeutic strategies in intestinal tube motility disorders.

Due to the lack of a full understanding of TC functionality, many authors have preferred to use the term ICC-like cells (ICC-LC). To highlight the role of TCs in cavity motility, it is necessary to study the extreme conditions achieved by the obstruction of the tubular organs in which they are found. There is a decrease in ICC-LC in obstructions [19], with a sharp decrease in areas with a significant muscle layer [20]. This signaling by mechanical obstructive stimuli can be correlated with the hypothesis of transforming TCs into SCs [21]. Moreover, intercellular fibrosis associated with decreased ICCs is associated with motility disorders [22]. This raises the question of whether there is a causal relationship between fibrosis and a decrease in mechanical stimulation, and consequently a decrease in TCS stimulation. The causal correlation seems to be confirmed by a close anatomical relationship between enteric fibrosis and ICC [23].

**Figure 1:**
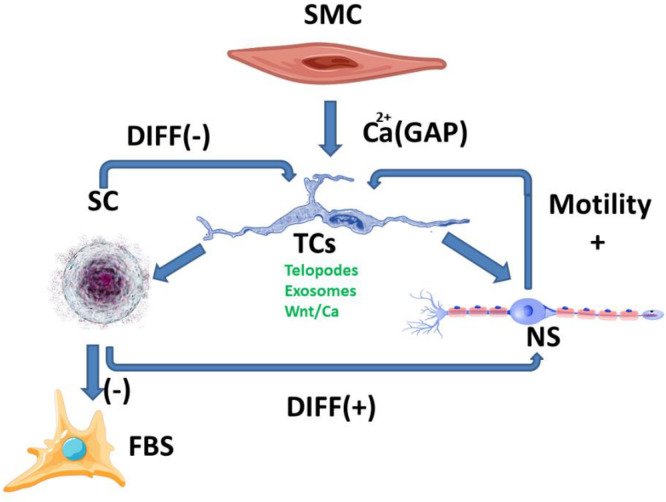
SC-stem cells, FBS-fibroblasts, NS-neurons, SMC-smooth muscle cell; Breaking the balance between the two ways of stimulating TCs (positive and negative) can lead to either an increase or local decrease in digestive motility, mediated by morphological and functional changes. These changes are currently highlighted at the level of fibrosis and/or neuronal plexus development, although the signaling pathways are much more complex.

The differentiated development of the myenteric and the submucosal plexus is suggestive of a mechanism regulating the function of the muscle mass situated in the immediate proximity of each plexus. The involvement of calcium channels with a degree of mechanosensitivity in signaling, which is induced by mechanical stimuli on telocytes [4] along with their ability to achieve gap junctions [5] with smooth muscle cells, can amplify signaling along the Wnt/Ca^2+^ pathway. Identifying Wnt signaling pathways in TCs [24] and TCs’ ability to modulate the functions of other cells through gap junctions or exosomes [25] paves the way towards multicellular signaling pathways which are correlated with the smooth muscle contraction of the digestive tube. Thus, stimulating neuronal development, directly or through SCs, can lead to positive feedback to maintain the function and development of smooth cell contractions. Stimulating SCs’ transformation in fibroblasts with a subsequent production of ECM can decrease their permeability for exosomes and cellular extension growth, and can lead to negative feedback signaling (Figure 1).

The balance between the different signaling pathways could lead to an understanding of telocytes’ behavior depending on environmental stimuli [21]. Maintaining the balance between the positive and negative feedback mechanisms can also be interpreted through the theoretical model’s extrapolation to inflammatory-type pathologies. This correlates with changes in digestive tube motility [17]. In addition, inflammation modulates the ECM permeability to telocytes exosomes.

Fibrosis can modulate tissue permeability for signaling factors and can be the result of several pathways such as ones involving mast cells [26,27], particularly due to the proximity between TCs and mast cells [28]. The involvement of mast cells in gastrointestinal smooth muscle homeostasis in both physiological and pathological processes [26] suggests that our proposed functional model is just one of the multiple signaling pathways which influence each other.

The involvement of the Wnt-β-catenin signaling pathway in stem cells’ behavior and the development of intestinal polyps [29] correlates with the proposed theoretic model and highlights the complexity of the signaling pathways. Telocytes seem to be involved differently in tegumentary fibrosis, intestinal inflammation, liver inflammation, and psoriasis [30]. However, the TCs’ differentiated functions depend on local factors and are probably due to the diversity and complexity of the interconnected signaling mechanisms. Wnt signaling pathways seem to restrain cell proliferation. They are involved in intestinal TCs’ functions and regenerative functions in CD [10,14]. Intestinal human mesenchyme remodeling [31,32] suggests a complex interplay in intercellular signaling with a limitative effect on cell proliferation. This might explain the relatively low incidence of GI inflammatory or motility disorders.

The Ca^2+^ signaling pathway may have several functions depending on the local cellular environment. One of them is modulating vascular smooth cell function directly through gap junctions [33]. This hints to an important link between the proposed functional model of TC signaling and vascular modulators which can influence tissue permeability for intercellular signaling factors. In addition to the functional aspects described, TCs have a direct influence on vascular structures by means of angiogenesis [34,35]. Through these functional and structural changes induced by TCs, new opportunities can arise for the partial testing of the proposed functional model of TC signaling in animal models by use of vasoactive medications, such as calcium function mediators.

## 4. Intestinal Colics as a Simplified Model for Understanding the Role of TCs in Digestive Motility

**Figure 2:**
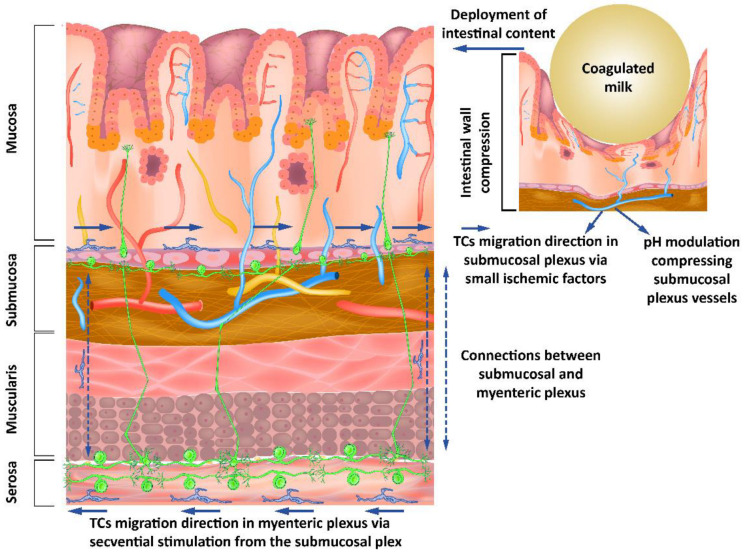
Submucous and myenteric nerve plexuses’ organization showing how modulating the pH as a response to mechanical stimuli from the intestinal lumen influences the neurons through the telocytes’ response to these stimuli.

TCs have been found in lamina propria, i.e., proximal to the lumen of the digestive tract [36]. Important changes in pH in the intestinal lumen have been found to translate to only minor changes in the juxta-mucosal area [37]. Although the changes are reduced in the mucosa by intrinsically changing the pH of the intestinal lumen, the presence of a bolus of materials in the digestive tract can lead to mucosal compression with a decrease in pH. This is done asymmetrically when compared to mechanical compression due to the occurrence of vasodilatory compensatory mechanisms. The outcome is an asymmetry in pH decrease when compared to mechanical compression. The resulting anisotropy allows signaling for the movement of the telocytes in the opposite direction of the fecal bolus movement. The phenomenon described occurs due to the compensating mechanisms that determine the presence of a higher pH gradient in front of the mechanical changes and a lower pH gradient behind (Figure 2).

TCs have the ability to signal via TGF-β1 [38] with morphological alterations. The ability of TCs to migrate predominantly into the nearby mucosal layer may be correlated with their ability to influence synaptogenesis through TGF-β1 [39]. Moreover, it has been shown that TGF-β1 increases neuronal excitability [40]. The ability of TGF-β to influence astrocyte-mediated synaptogenesis [41], which can act as a scavenger mechanism positively influencing neuronal reorganization, or as a neuroprotector for the preservation of neuronal architecture, creates the premise of modulation between TCs and neurons in the intestinal tract. Lesions that lead to deprivation of stimuli may determine a decrease in TGF-β and TGF-β1 due to oligodendrocytes, but not to astrocytes and microglia [42]. Due to the ICC-LC age-dependent response to obstruction in cavity organs [43], it is important to note that the response is maximal in the first year of life if quantified by the number of these cells. The first year of life has, with regard to the aforementioned aspects, a significant advantage given by the uniformity of the contents of the intestinal tract as achieved by breastfeeding. Maternal milk has a genetic determinism with a high degree of uniformity and relatively small changes depending on external factors, compared to adult diets.

Interrelations between neurons, their supporting cells, and TCs suggest a distal-to-proximal organization of telomeres in the submucosal space, with a similar orientation of neurons through modulatory mechanisms of synaptogenesis. In the present model, the existence of the myenteric nervous plexus near the submucosal nervous plexus, with diffuse connections between them, creates an opportunity for organizing the myenteric plexus in the opposite direction due to the synaptic plasticity considerations [44]. 

**Figure 3:**
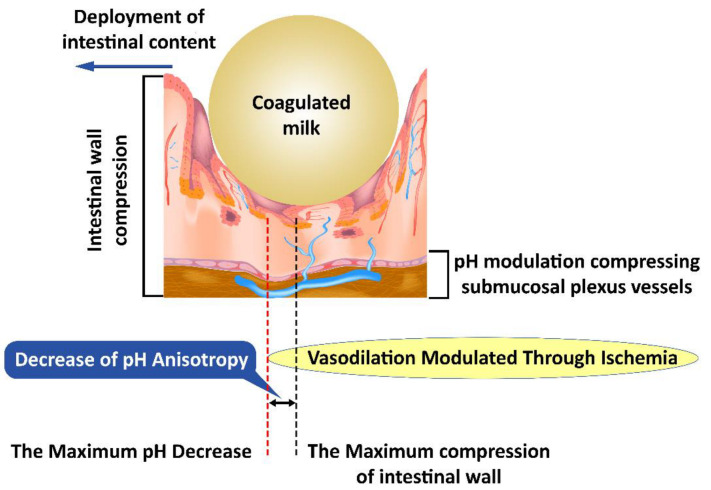
Mechanical compression induces anisotropy of pH changes; first submucosal vessels are affected and subsequently the pH-dependent vascular compensatory mechanisms.

Our hypothesis explores the differences between the peak of the pH changes and the peak of the mechanical changes in pH anisotropy (Figure 3) as a representation of a theoretical model that can be evaluated from the perspective of the presented literature. Consistent changes in the fecal bolus may lead to a decrease in intestinal mucosal compression, resulting in a decrease in pH changes, as well as a decrease in anisotropy. This is explained by the existence of pH-dependent compensatory vascular mechanisms. Thus, a significant decrease in pH increases the importance of compensatory mechanisms, but a low pH decrease implies a reduced weight of these mechanisms. On the one hand, the pH drop peak is close to the peak of the mechanical changes, and on the other hand, the pH decrease and increase slopes flatten with decreasing fecal bowl consistency. A decrease in fecal bowel consistency will lead to a decrease in the anisotropy of pH changes mediated by the decrease in mechanical compression and pH-dependent vascular compensatory mechanisms.

Changes induced by pH anisotropy are sufficiently sensitive to be modulated by bacterial flora in the intestinal lumen. This may explain the need to have a uniformly distributed flora along the digestive tract, although conditions vary slightly from one area to another. The decrease in colonic microbiotic dysplasia [45] presents itself as a specific marker and creates the opportunity to specifically identify the need for bacterial strains that have not developed sufficiently with possible therapeutic implications. Intestinal colic, however, is not an aggressive pathology with a specific treatment [46]. This opportunity seems to be confirmed by the existence of pH-based bacterial screening mechanisms [47] as well as by the existence of distinct pH receptors for Helicobacter pylori [48], which may be possible for other intestinal bacteria. The bacterial selection along the intestinal tract leads to striking a balance between the various bacterial clones, the environmental conditions, and the fecal bowl consistency (Figure 3).

The theoretical model proposed to explain intestinal colic allows us to understand the interaction between TCs, surrounding tissues, and microbes. In addition, it opens a window toward understanding the pathologies that affect the normal functionality of TCs and their modulating role in morphogenesis. 

Due to the limited spatial changes in the reduction in the number of bacterial clones in intestinal colic, we can extrapolate the information that TCs communicate via extracellular vectors, and consequently, they are modulated by the different locally available membrane lipids. The effect of intestinal bacteria is outlined not only as an intestinal bolus with a spatially-appropriate mechanical consistency varying with location along the intestinal tract, but also as a lipid source that can alter membrane fluidity and the processes that depend on this fluidity. For example, TCs could modify the response to channel-mediated mechanical stimulation [4], while also modifying TCS-specific exocytosis and endocytosis. 

**Figure 4:**
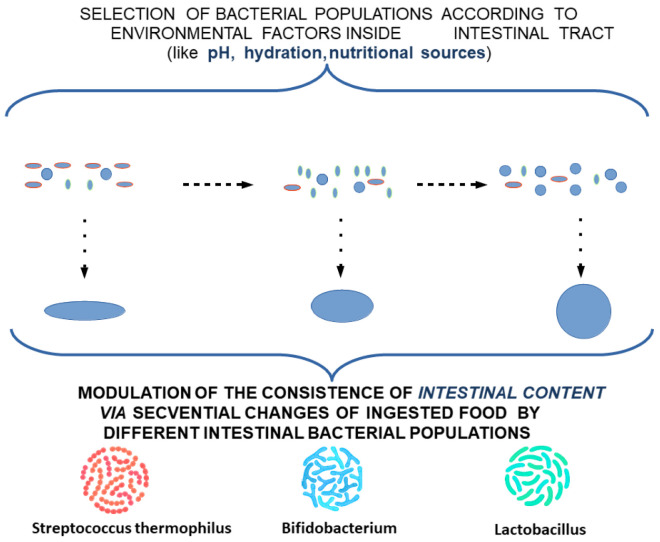
Modulation of the consistency of intestinal content by different intestinal bacterial populations.

An explanation of the pattern of intestinal colic is outlined as a punctual decrease in the rate of migration of TCs in the submucosa, which can lead to a consecutive decrease in the myenteric plexus. This theoretical model would explain a punctual decrease in the rate of fecal bolus movement, and consequently a slight distension and colic-specific pain. The occurence of punctual localizations would explain the relatively consistent association with the time of food intake and would provide insight into the disappearance of local dysmotility and associated symptoms by the action of the unaffected proximal muscles (Figure 4).

**Figure 5:**
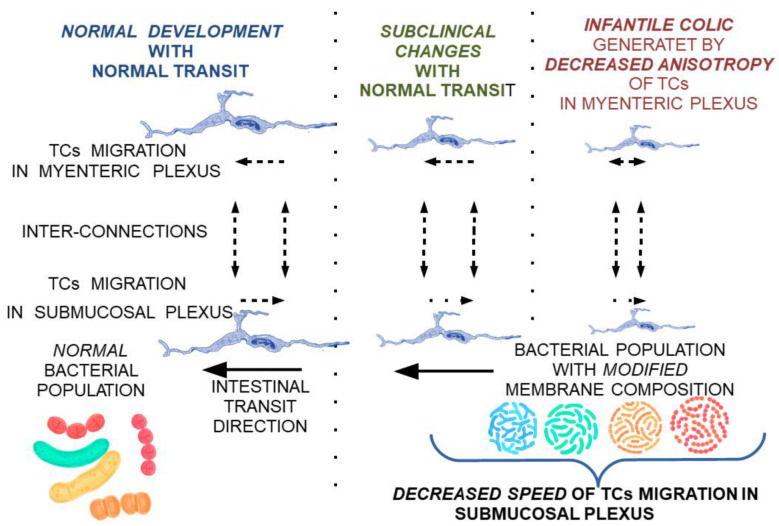
Theoretical model on the reversible progression of maturation changes of the submucosal and myenteric nerve plexus under the influence of TCs modulated by membrane fluidity changes secondary to bacterial populations with a membrane lipid composition not adapted to local conditions.

**Figure 6:**
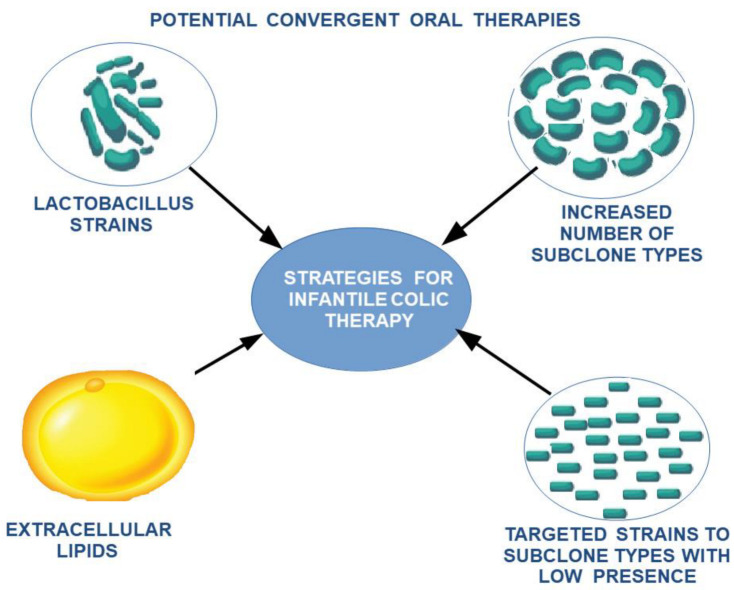
The proposed therapeutic strategy for the reduction in intestinal colic.

This can explain the need for the adaptation of intestinal flora with the transition from an exclusive diet to a rich diet, which is also associated with changes in the maturation of intestinal structures according to new environmental conditions. To alleviate the stress factors affecting the child and the family, and to ease the transition of intestinal flora and intestinal tract adaptation to these changes, a potential treatment using a combination of different lactobacillus subclones with a high clonal diversity—highlighting missing subclones according to age and food supplementation—can be proposed. Due to the variation along the digestive tract in the optimum conditions for each cell subclone, extracellular lipid feeding can be supplemented as a feasible measure to oppose the low viability of cell clones induced by food intake (Figure 5 and Figure 6).

## 5. CD, UC, and Irritable Colon as Models of Validation of the Proposed Theoretical Model

To validate the theoretical model of the involvement of TCs in the maturation and functioning of the intestinal nerve plexus, the choice of these three pathologies, which are relatively different from each other but have diarrhea as a common element, shows promise. CD accompanies a discontinuous network of TCs [2]. This can be explained by a decrease in their number through accelerated migration from a certain area, or by a reduced intake in distal areas. Due to the associated diarrhea, it can be extrapolated that the function of the TC network in the myenteric plexus is appropriate or even accelerated. 

**Figure 7:**
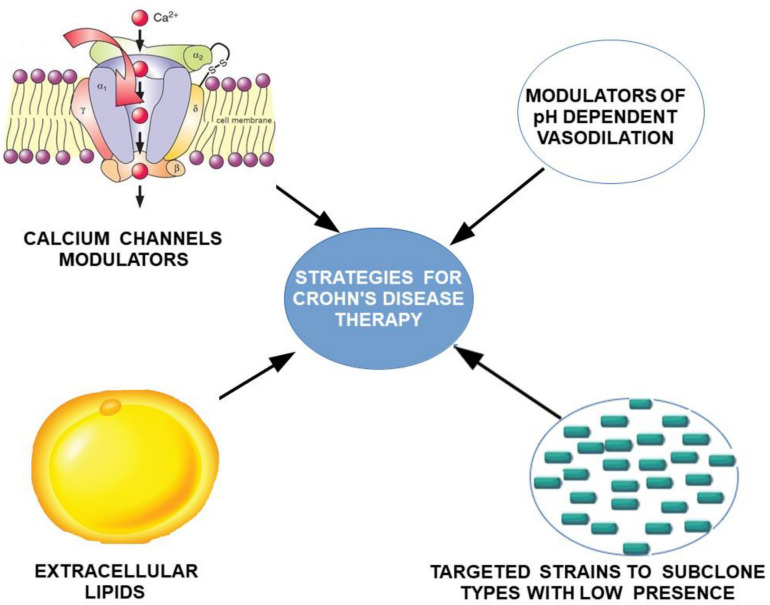
Proposed strategy for treating Crohn’s disease by targeting dependent and pH-independent vascular hyperreactivity, and by direct targeting of TCs migration.

This concentration of the initial changes in the submucosal plexus, with the existence of ulcerative lesions, suggests the existence of impaired pH-dependent vasodilator reflexes, which lead to the acceleration of local TC migration, but also to the occurrence of ischemic ulcers. The local character of the lesions, with the presence of healthy areas between affected ones, suggests the involvement of a genetic mosaicism. Based on our theoretical model for the explanation of Crohn’s disease, potential therapeutic targets can be identified by attempting to manipulate TCs through clonal strains and extracellular lipids to decrease the rate of migration of TCs into the submucosal plexus. Additionally, vascular targeting by direct modulators of pH-mediated vasoconstrictive reflexes or indirectly by calcium channel modulators could result in effective ulcerative and diarrheal treatment via modulation of TCs by aligning the rate of migration of TCs to the submucosal layer (Figure 7).

UC is defined by the contiguity of lesions without healthy areas between afflicted areas. According to the proposed theoretical model, lesions are not centered on the pathophysiological vascular component. It is possible to extrapolate a decrease in TCs by exhausting distal reserves (from the rectum) through the proximal migration process. Ulcerative lesions could be explained by a protective relationship between TCs and the vascular bed. This mechanism is incompletely understood. There is probably a TC-induced vasopressor tone, in the absence of which the distal vessels fail to maintain their integrity. This could be explained by the importance of calcium channel signaling [4] and the proximity of TCs to the vascular system [49].

If the described hypothesis is confirmed, an auto synthesis of TCs would be required as a rectal cell reservoir for the initial treatment of lesions. While the suggested treatment may only be provisional, if confirmed to be effective, it could open up new therapeutic opportunities for the recirculation of TCs from the myenteric plexus to the submucosal plexus. If the aforementioned secondary mechanism is deficient, a stimulation of TC migration to the intestinal lumen could be achieved by increasing the response to mechanical stimuli [4] by modulating membrane fluidity through the ingestion of lipids that can alter membrane fluidity. This second therapeutic proposal could slow down the proximal progression and possibly lead to the slow reversibility of the lesions. The two therapeutic strategies have not been tested thus far and could be a turning point in understanding the role of TCs in responding to environmental factors.

The irritable bowel can be interpreted in terms of the proposed theoretical model if we consider the apparent correlation between colic and migraines [50]. Due to the fact that one of the major causes of migraine is of vascular origin, we could extrapolate the existence of a mechanism of vascular hyperreactivity. If so, we propose as a potential treatment the use of specific modulators of pH-dependent vasodilation. Unlike the previous two pathologies, the irritable colon has morphological changes classified as subclinical (submucosa only). In this case, TCs modulate nerve plexus function but without significant histological changes. It may be possible to use membrane fluidity modulators as a complementary treatment.

In all three of the proposed pathologies, possible non-correlations with the proposed theoretical model could be explained by a relatively small number of modifiable variables. This suggests a high probability of the validity of the proposed theoretical model.

## 6. Conclusions

The theoretical model of migrating TCs into the submucosal layer in the anti-peristaltic direction and TCs in the peristaltic myenteric plexus layer with a possible reintroduction of TCs from the myenteric to the submucosal layer could explain a significant number of disorders which have thus far been incompletely understood. Developing from our model, a significant number of relatively easy-to-use therapeutic strategies allow for the validation or invalidation of its various components. Even a partial confirmation of the described theoretical model could explain the importance of TCs in interacting with the environment and the in-vitro characterization difficulties of their functions. The simplified model of the digestive tract, which shows relatively small changes in one area compared to another adjacent area, creates the opportunity to understand the complex mechanisms of interaction with the environment. In confirming their ability to modulate nerve plexus function at this level, a new stage opens in understanding the functioning of TCs’ interactions with neurons in areas with higher structural and functional complexities.

## Data Availability

Not applicable.
